# Survival patterns of neonates born to adolescent mothers and the effect of pregnancy intentions and marital status on newborn survival in Kenya, Uganda, and Tanzania, 2014–2016

**DOI:** 10.1080/16549716.2022.2101731

**Published:** 2022-08-26

**Authors:** Malachi Ochieng Arunda, Anette Agardh, Markus Larsson, Benedict Oppong Asamoah

**Affiliations:** Social Medicine and Global Health, Department of Clinical Sciences, Lund University, Malmö, Sweden

**Keywords:** Adolescents, survival analysis, unintended pregnancy, neonatal mortality, marital status, East Africa

## Abstract

**Background:**

Adolescent pregnancy and associated neonatal mortality are major global health challenges. In low-income settings where 90% of the 21 million global adolescent pregnancies occur, half are unintended and a fifth experience unsafe abortion. In Kenya, Uganda, and Tanzania, the survival patterns of neonates born to adolescents are unclear.

**Objectives:**

To assess survival patterns among neonates born to adolescents and the effect of pregnancy intentions and marital status on survival in Kenya, Uganda, and Tanzania.

**Methods:**

Cross-sectional data from demographic and health surveys in Kenya, Uganda, and Tanzania 2014–2016 were used. Kaplan-Meier estimates investigated patterns of neonatal survival among adolescent mothers, aged 15–19 years, compared to mothers aged 20–29 years. Cox proportional hazards regression determined the hazard ratios (HR) for the predictors of neonatal survival.

**Results:**

About 50% of adolescent pregnancies were unintended and neonatal death rate was twice as high than older mothers (26.6 versus 12.0 deaths/1000 live births). The median survival time was two days for adolescent-born babies and four days among older mothers. The hazard of death for all adolescent-born neonates was about twofold that of 20–29 years-old-mothers, HR 1.80 (95% CI 1.22–2.63). Among married adolescents with unintended newborn pregnancies, the HR was 4-folds higher than corresponding older mothers, HR 4.08 (95% CI 1.62–10.31). Among married, primiparous adolescents with unintended pregnancies, the HR was six times higher than corresponding older mothers.

**Conclusion:**

Our findings reveal how unintended pregnancies and deaths of neonates born to adolescents contribute substantially to preventable neonatal deaths in East Africa. Full implementation of existing adolescent health policies and utilization of contraceptives should be ensured. Partnership with youths and novel efforts that address sociocultural norms to reduce adolescent pregnancies or marriage should be supported. Regulations requiring adolescents’ obstetric care conducted by only skilled personnel should be introduced and implemented.

## Background

Adolescent pregnancy and associated neonatal mortality are major global health burdens [1]. It is estimated that every year, 21 million pregnancies occur among girls aged 15–19 years. In 2018, approximately 12 million adolescents gave birth [2] with the birth rates ranging between 12 and 97 births per 1000 adolescent girls in high- and low-income countries, respectively [3]. The World Health Organization (WHO) estimates that in low- and middle-income countries (LMIC) where over 90% of global adolescent pregnancies occur each year [1], half of them are unintended [1,2]. In 2019, 2.4 million newborns died in their first four weeks after birth (neonatal period) [4], and the leading causes (risk factors) for these deaths included infections, prematurity, and birth complications [4]. Neonates born to adolescent mothers are known to be at the highest risk for these major risk factors of neonatal deaths, as compared to newborns to older mothers aged 20–34 [3,5,6]. However, the proportions and patterns of deaths among neonates born to adolescent mothers compared to neonatal deaths among older mothers are unclear.

Adolescent age is a period characterized by rapid growth and development. Both the height and weight of the body increase substantially. Adolescent girls can gain an average of up to 25 kilograms in weight and up to 20 centimeters in height [7]. For a pregnant adolescent, competition for nutrients that arises between the fetus and the mother can lead to increased risk for complications, such as low birthweight, prematurity, and perinatal morbidity and mortality [8]. These complications are exacerbated in low-income settings where adolescents can be at risk of malnutrition due to food shortages.

In certain high-income countries (HIC), such as Sweden, adolescent pregnancy is not a major burden mainly due to constant efforts that are made to minimize sexually risky behaviors through sexual and reproductive health education and access to contraceptives [9]. In LMIC, where adolescent pregnancy rates are highest, over 30% marry before 18 years of age [3]. Along with limited knowledge and access to contraceptives, this is mostly due to societal pressure, sexual coercion, poverty, lack of access or motivation in education, and early childbearing [10]. Child marriage is a leading risk factor driving adolescent pregnancy [1,10,11] and the highest levels of these marriages are in sub-Saharan Africa (SSA) [12]. A recent meta-analysis by Kassa et al. that estimated the pooled prevalence of adolescent pregnancy in SSA indicated that East African countries had the highest prevalence (21.5%) in the region [13] but also globally [14]. Most adolescent pregnancies in SSA result in severe health consequences, maternal and neonatal mortalities, increase in school dropout, and far-reaching socioeconomic impacts on individuals and societies [1,15].

In Kenya, Uganda, and Tanzania where neonatal death rates have been persistently high at 20–22 per 1000 live births between 2014–2020 [16–18], very few studies have investigated the neonatal survival patterns (time-to neonatal-death patterns) among neonates born to adolescent mothers as compared to older mothers. Studies in the East African Community have found higher risks of neonatal deaths among adolescent girls compared to older mothers [19–21]. However, all these studies modelled neonatal death as a one-time event and no studies to our knowledge examined the newborn survival pattern over time during the neonatal period for adolescent mothers.

Elsewhere in southern Asia, studies have found significantly higher odds of neonatal deaths among mothers (of all ages) whose newborn pregnancies were unintended (unwanted or mistimed) compared to intended pregnancies [22,23]. Such studies are rare in SSA and almost none among adolescents. A 2020 study on factors associated with unintended pregnancies among all mothers of reproductive age (15–49 years) in SSA reviewed about 29 studies but none reported on neonatal mortality outcomes [24]. Nonetheless, in 2019, WHO citing Darroch et al. reported that full avoidance of unintended pregnancy through contraceptives and full provision of maternal and newborn care would reduce global neonatal deaths by 80% per year [25,26].

Marital status, also known to be a determinant of neonatal survival [27], has not been adequately investigated among adolescents in SSA. Our previous studies in East Africa found a higher proportion of low birthweight babies and neonatal deaths among adolescent and young mothers below 24 years of age but these findings were inconclusive and required further investigations [28,29]. Furthermore, to achieve the second target of the third global sustainable development goal (SDG 3), which aims to reduce neonatal deaths by at least as low as 12 per 1000 live births by 2030 [30], research on neonatal survival patterns (time-to-death of neonate) among adolescent mothers is critical. This study aims to examine neonatal survival patterns among neonates born to adolescent mothers aged 15–19 years and the effect of pregnancy intentions and marital status on mortality patterns in Kenya, Uganda, and Tanzania. We utilized older mothers aged 20–29 years for comparison. The findings could expose aspects of neonatal survival among adolescents that could have implications for prioritization and allocation of resources to effectively reduce adolescent pregnancies and overall neonatal deaths in the three East African countries.

## Methods

### Study setting

Kenya, Uganda, and Tanzania are three LMIC in the East African Community with an estimated total population of 140 million and sex ratio of about 1:1 (31–33). Over 70% of the population live in rural areas with farming as their main economic activity [31–33]. Adolescents aged 15–19 years constitute about 20% of the total population in East Africa (EA) and about half (15 million) are girls [34–36]. The prevalence of adolescent pregnancy in EA is about 21% [13], and over 30% of girls in East and Southern Africa marry before 18 years of age [37]. These three countries are among 20 countries that contribute the highest neonatal deaths globally [38]. Their neonatal death rates range from 20–22 deaths per 1000 live births [16–18]. These countries are very similar in their maternal, adolescent and neonatal health situations, policies, and are all in their pathways towards achieving universal health coverage.

### Data source and study design

We used secondary data from nationally representative, demographic, and health surveys’ (DHS) data pooled from Kenya (2014), Uganda, and Tanzania (2015–2016). The DHS primary data collectors, collect nationwide household, health, reproductive, and mortality data using a cross-sectional design. To minimize recall bias, we used data for the most recent live-born, singleton neonates born to adolescent mothers 15–19 years old. For comparison, corresponding mothers 20–29 years old were also included in the study data and used as a reference. These were maternal ages at the time of the DHS interviews. Also, we included only children born within 1–59 months (~5 years) prior to the 2014 and 2015–2016 DHS surveys in the respective countries. We utilized data for 18,248 neonates born within five years preceding the commencement of DHS data collection, samples, 8557, 5910, and 3781 from Kenya, Uganda, and Tanzania respectively. A written request was sent to the DHS secretariat and permission was obtained to use the datasets. The DHS Program has been mandated by host countries to collect health data for purposes of research to improve maternal and newborn health. As a standard procedure, the DHS Program obtained ethical consent from all participants and the ethical approvals from the country or institutional review boards. The DHS data collection procedure adheres to national and international ethical requirements for research involving human subjects. More details on DHS survey instruments and methodology can be obtained from: https://dhsprogram.com/methodology/Survey-Types/DHS-Methodology.cfm

### Study variables

#### Outcome and predictor variables

Neonatal mortality (newborn death within 28 days after birth) was the outcome event of interest. Maternal adolescents aged 15–19 years were the predictor variable with the older mothers aged 20–29 years as the reference age group. Stratified models were used to determine the effects of marital status and pregnancy intentions on neonatal survival for adolescent mothers, as compared to the corresponding mothers in the older age-group.

#### Explanatory variables

These constituted confounding variables that have been associated with either adolescent pregnancy or neonatal mortality and morbidity. They included sociodemographic factors, as well as maternal health care and newborn factors. Maternal education level is known to influence neonatal well-being [39]. This was dichotomized into no education/primary and secondary/higher education, respectively. Poor economic (wealth) status has also been linked to neonatal mortality [40], and was categorized into poor, middle, and rich. The wealth status was computed based on living standards considering family assets and access to water and sanitation facilities. Place of residence, particularly rural (remote) and urban slum residency, has also been associated with neonatal deaths compared to urban non-slum areas [41,42]. Place of residence was categorized as rural and urban. Sex of child [43] was categorized as male or female, and low birthweight (lbw) categorized as `yes´ to mean <2500 g and `no´ for none lbw (≥ 2500 g) [44]. As part of the study objectives, marital status and pregnancy intentions were also hypothesized to impact neonatal survival among adolescent mothers. Pregnancy intention was grouped as intended or unintended. These were further dichotomized into married if currently married, and single if never married, divorced/separated or widowed. Antenatal- and postnatal care visits and health facility delivery are known to reduce the risk of neonatal morbidity and death [45,46]. These were also adjusted for in the analysis model according to the WHO recommendations that applied at the time of data collection. Additionally, other variables associated with adolescent pregnancy, i.e. use/access to, and decision making for, use of modern contraceptives [47,48] and age at first sexual intercourse were also included in the study [49].

## Data analysis

We also used chi square tests to examine the distribution of sociodemographic, maternal, and newborn variables between adolescent mothers 15–19 years old and mothers aged 20–29 years at significance level, *p* < 0.05.

The Kaplan-Meier method [50] was used to estimate the visual patterns of survival of neonates during 28 days after birth. The survival time was right censored since deaths continue to occur beyond this neonatal period. In the survival analysis (or time-to-death analysis) in the study context, survival meant remaining free from death over the neonatal period of 28 days, and the time of origin was the time/day a baby was born alive. The neonate status after 28 days was dichotomized into dead or alive (or missing). The endpoint of neonate was death, neonates whose survival information was missing or lived after 28 days were censored. We analyzed not only the numbers of neonates who died but also the times-to-death for the neonates born to adolescents, compared to those born to older mothers, and all these provided us with neonatal survival pattern. The log-rank method was used to assess the equality of the survival curves.

Multivariate analysis was conducted using Cox proportional hazards regression to assess the hazard of death among neonates born to adolescents versus those born to mothers 20–29 years old, while adjusting for other risk factors. Stratified analyses of marital status and newborn pregnancy intentions were also executed. Both crude and adjusted hazard ratios were obtained at 95% confidence interval (95% CI). The proportional hazard assumptions were assessed using both global test and the log-log transformation to the survival function. We utilized Stata analytical software version 16 [51].

## Results

Table 1 indicates that, overall, about 50% of all adolescent mothers, 15–19 years old, had their first sexual encounter at 15 years old or below compared to 28% among older mothers, 20–29 years old, in Kenya, Uganda and Tanzania. However, there was a slightly higher proportion of early sexual debut among those 15 years or below in Tanzania (59%) compared to Kenya (48%). Despite over 63% of adolescent mothers being married in all countries combined, about half of all pregnancies among adolescents were unintended. For Tanzania, a much higher proportion (61%) of adolescent pregnancies were intended, comparable to their older counterparts (66.7%) in Tanzania and these were statistically significant (p < 0.05). More than three-quarters (76.5%) of adolescent mothers in Kenya, Uganda and Tanzania lived in rural areas, as compared to 69% of older mothers.

**Table 1.** Distribution of study variables by adolescent mothers (≤ 19 years old) and mothers 20–29 years old, in Kenya, **Uganda** and **Tanzania**, 2014–2016.

	Overall			Kenya			Uganda			Tanzania		
Variables	15–19 years,n = 2255	20–29 years,n = 15,993	*X^2^*, P value (95%CI)	15–19 years,n = 883	20–29 years,n = 7674	*X^2,^* P value (95%CI)	15–19 years,n = 825	20–29 years,n = 5085	*X^2,^* P value (95%CI)	15–19 years,n = 547	20–29 years,n = 3234	*X^2,^* P value (95%CI)
	n (%)	n (%)	95%CI	n (%)	n (%)	95%CI	n (%)	n (%)	95%CI	n (%)	n (%)	95%CI
**Marital status**												
Single	821(36.5)	2730(17.3)	0.001	365(41.5)	1223 (16.2)	0.001	275(33.5)	876(17.4)	0.001	181(33.1)	631(19.7)	0.001
Married	1427(63.5)	13,088(82.7)		514(53.5)	6337(83.8)		547(66.5)	4173(82.6)		366(66.9)	2578(80.3)	
**Newborn pregnancy intended**												
Intended	884 (49.6)	7534(62.8)	0.001	177(43.2)	2378(64.7)	0.001	371(45.0)	3000(59.0)	0.001	366(61.4)	2156(66.7)	0.017
Unintended (unwanted/mistimed)	898(50.4)	4461(37.2)		233(56.8)	1298(35.3)		454(55.0)	2085(41.0)		211(38.6)	1078(33.3)	
**Education level**												
No education	178(7.9)	2209(14.7)	0.001	77(8.8)	1350(19.1)	0.001	31(3.8)	353(7.4)	0.001	70(12.8)	506(15.8)	0.07
Primary/secondary or higher education	2070(92.1)	12,827(85.3)		802(91.2)	5738(80.9)		791(96.2)	4395(92.6)		477(87.2)	2694(84.2)	
**Place of residence**												
Urban	530(25.5)	4890(30.6)	0.001	283(32.1)	2868(37.4)	0.002	129(15.6)	1119(22.0)	0.001	118(21.6)	903(27.9)	0.002
Rural	1725(76.5)	11,103(69.4)		600(67.9)	4806(62.6)		696(84.4)	3966(78.0)		429(78.4)	2331(72.1)	
**Wealth Status**												
Poor	1217(54.0)	7275(45.5)	0.001	490(55.5)	3797(49.5)	0.001	451(54.7)	2251(44.3)	0.001	276(5.4)	1227(37.9)	0.001
Middle	445(19.7)	2905(18.2)		192(21.7)	1319(17.2)		147(17.8)	945(18.6)		106(19.4)	641(19.8)	
Rich	593(26.3)	5813(36.4)		201(22.8)	2558(33.3)		227(27.5)	1889(37.1)		165(30.2)	1366(42.2)	
CI – Confidence interval. Primiparous – first time mothers, Multiparous – Given birth at least once previously. *X^2^ – Chi square*												
**Decision maker for using contraceptives**												
Mainly respondent	1031(24.1)	61(18.5)	0.02	18(20.1)	442(27.1)	0.200	24(20.7)	421(25.8)	0.178	9(11.7)	168(16.6)	0.458
Mainly husband/ partner, others	350(8.2)	39(11.8)		16(18.0)	187(11.5)		20(12.2)	131(8.1)		3(3.9)	27(2.6)	
Joint decision	894(67.7)	230(69.7)		55(61.9)	1000(61.4)		110(67.1)	1075(66.1)		65(84.4)	819(80.8)	
**Modern contraceptive use**												
Yes	464(20.6)	4684(29.3)	0.001	134(15.2)	1810(23.6)	0.001	215(26.1)	1787(35.1)	0.001	115(21.0)	1087(33.6)	0.001
No	1791(79.4)	11,309(70.7)		749(84.8)	5864(76.4)		610(73.9)	3298(64.9)		432(79.0)	2147(66.4)	
**Age at first sexual encounter**												
<15 years	1176(52.2)	4543(28.4)	0.001	421(47.7)	1915(25.0)	0.001	431(52.2)	1655(32.6)	0.001	324(59.2)	973(30.1)	0.001
16–18 years	909(40.3)	6401(40.0)		296(33.5)	2324(30.3)		391(47.4)	2524(49.6)		222(40.6)	1553(48.0)	
19–28 years	5(0.2)	2864(17.9)		1(0.1)	1252(16.3)		3(0.4)	904(17.8)		1(0.2)	708(21.9)	
At first union	165(7.3)	2185(13.7)		165(18.7)	2183(28.4)		–	2 (0.1)		–	–	
**Parity**												
Primiparous	964(42.8)	10,563(66.0)	0.001	391(44.3)	5229(68.2)	0.001	347(42.1)	3235(63.7)	0.001	226(41.3)	2099(64.9)	0.001
Multiparous (≥1)	1291(57.2)	5430(34.0)		491(55.7)	2443(31.8)		477(57.9)	1846(36.3)		321(58.7)	1134(35.1)	
CI – Confidence interval. Primiparous – first time mothers, Multiparous – Given birth at least once previously. *X^2^ – Chi square*												

Table 2 shows the distribution of study variables by censored and neonatal deaths for all mothers aged 15–29 years old in Kenya, Uganda and Tanzania. The neonatal mortality rate (NMR) was two times higher (26.6 versus 12.0 deaths per 1000 live births) among adolescents than among older mothers. Newborn sex, antenatal care visits, postnatal care visits, wealth status, parity, birthweight, and marital status indicated associations with neonatal survival status (*p* < 0.05, from chi square test) across these sub-populations. Country-specific findings show that the statistical significance for the study variables against neonatal survival outcomes slightly varied, but parity and postnatal care attendance indicated statistical significance with neonatal survival in all three countries (p < 0.05).

**Table 2.** Distribution of study variables by neonatal survival status among adolescents and mothers aged 20–29 years in Kenya, Uganda and Tanzania, 2014–2016.

	**Overall**, N = 18,024			**Kenya**, n = 8459			**Uganda**, n = 5834			**Tanzania**, n = 3732		
	Censored	Died	*X^2^*, P value (95%CI)	Censored	Died	P value (95%CI)	Censored	Died	*X^2^*, P value (95%CI)	Censored	Died	*X^2,^* P value (95%CI)
**Maternal age**												
^a^Adolescents, 15–19 years	2160(12.2)	59(23.8)	0.001	867(10.4)	9(9.6)	0.803	778(13.6)	30(31.3)	0.001	515(14.0)	20(34.5)	0.001
^b^20–29 years	15,616(87.8)	189(76.2)		7498(89.6)	85(90.4)		4960(86.4)	66(68.8)		3158(86.0)	38(65.5)	
**Marital Status**												
Single (unmarried)	3443(19.6)	60(24.7)	0.046	1553(18.8)	17(18.9)	0.986	1116(19.6)	20(21.0)	0.719	774(21.2)	23(39.7)	0.001
Married	14,159(80.4)	183(75.3)		6700(81.2)	73(81.1)		4585(80.4)	75(79.0)		2874(78.8)	35(60.3)	
**Newborn pregnancy intentions**												
Intended	8173(61.0)	126(64.3)	0.345	2491(65.2)	26(66.7)	0.846	3261(58.5)	64(68.1)	0.060	2421(66.6)	36(63.2)	0.586
Unintended	5231(39.0)	70(35.7)		1331(34.8)	13(33.3)		2317(41.5)	30(31.9)		1215(33.4)	21(36.8)	
**Place of residence**												
Rural	12,504(70.3)	175(70.6)	0.939	5295(63.3)	59(62.8)	0.915	4523(78.8)	77(80.2)	0.742	2686(73.1)	39(67.2)	0.316
Urban	5272(29.7)	73(29.4)		3070(36.7)	35(37.2)		1215(21.2)	19(19.8)		987(26.9)	19(32.8)	
**Education level**												
No or primary education	12,485(70.2)	181(73.0)	0.347	5737(68.6)	71(75.5)	0.149	3968(69.2)	65(67.7)	0.761	2780(75.7)	45(77.6)	0.738
Secondary or higher	5291(29.8)	67(27.0)		2628(31.4)	23(24.5)		1770(30.9)	31(32.3)		893(24.3)	13(22.4)	
**Wealth status**												
Poor	8287(46.6)	112(45.2)	0.042	4194(50.1)	49(52.1)	0.085	2622(45.7)	46(47.9)	0.614	1471(40.1)	17(29.3)	0.105
Middle	3236(18.2)	60(24.2)		1466(17.5)	23(24.5)		1056(18.4)	20(20.8)		714(19.4)	17(29.3)	
Rich	6253(35.2)	76(30.7)		2705(32.3)	22(23.4)		2060(35.9)	30(31.3)		1488(40.5)	24(41.4)	
**Sex of newborn**												
Male	9084(51.1)	150(60.5)	0.003	4285(51.2)	58(61.7)	0.043	2961(51.6)	58(60.4)	0.087	1838(50)	34(58.6)	0.195
Female	8692(48.9)	98(39.5)		4080(48.8)	36(38.3)		2777(48.4)	38(39.6)		1835()	24(41.4)	
**ANC visits**												
<4	7770 (43.9)	138(55.7)	0.001	3814(45.7)	50(53.2)	0.149	2155(37.7)	51(53.0)	0.002	1801(49.3)	37(63.8)	0.028
≥4	9945(56.1)	110(44.4)		4524(54.3)	44(46.8)		3568(62.3)	45(47.0)		1853(50.7)	21(36.2)	
**Place of delivery**												
Home	5512(31.0)	69(28.0)	0.315	3140(37.6)	34(37)	0.900	1222(21.3)	20(20.8)	0.912	1150(31.3)	15(25.9)	0.374
Health facility	12,251(69.0)	177(72.0)		5212(62.4)	58(63.0)		4516(78.7)	76(79.2)		2523(68.7)	43(74.1)	
**PNC visit within 28 days after birth**												
Yes	4424(26.0)	29(11.7)	0.001	2250(27.8)	14(14.9)	0.005	1094(19.9)	7(7.3)	0.002	1080(31.5)	8(13.8)	0.004
No	12,595(74.0)	219(88.3)		5841(72.2)	80(75.1)		4405(80.1)	89(92.7)		2349(68.5)	50(86.2)	
**Low birthweight**												
Yes	1989(12.7)	62 (29.7)	0.001	305(12.4)	5(26.3)	0.068	662(17.9)	19(44.2)	0.001	312(13.6)	16(47.1)	0.001
No	13,685(87.3)	147(70.3)		2150(87.6)	14(73.7)		3036(86.1)	24(55.8)		2040(86.4)	18(52.9)	
**Parity**												
Primiparous	11,273 (63.4)	92(37.1)	0.001	5508(65.9)	41(44.1)	0.001	3500(61.1)	30(31.1)	0.001	2265(61.7)	21(36.2)	0.001
Multiparous (≥1)	6503(36.6)	155 (62.9)		2855(34.1)	52(55.9)		2233(39.0)	66(68.8)		1407(38.3)	37(63.8)	

^a^Neonatal mortality rate (NMR) = 26.6 per 1000 live births, ^b^Neonatal mortality rate (NMR) = 12.0 per 1000 live births, *X^2^ – Chi square*

LBW – Low birthweight, NBW -Normal birthweight. CI-Confidence interval

Primiparous – first time mothers, Multiparous – Given birth at least once previously

Table 3 shows the log rank estimates of the neonatal survival functions for adolescent mothers and mothers 20–29 years old, both overall and stratified by marital status or pregnancy intentions, or both. It indicates significantly shorter time-to-death for neonates born to adolescent mothers. Further stratification by marital status or pregnancy intentions shows similar findings for married mothers (*p*=0.0007), and for mothers who had (*p*=0.0001), or did not have (*p*=0.0035), intentions for the newborn pregnancies.

**Table 3.** Log rank estimates of neonatal survival functions between adolescent mothers and mothers 20–29 years old in Kenya, Uganda and Tanzania, 2014–2016, overall and stratified by marital status and/or pregnancy intentions and parity.

Groups and subgroups	Adolescents 15–19 years old		Mothers, 20–29 years		Log rank, *P* values (95% CI)
	Total number of live births	Deaths	Total number of live births	Deaths	
Overall	2219	59	15,805	189	0.0003
**Marital status**					
Married	1401	37	12,941	146	0.0007
Single (Unmarried)	812	21	2691	39	0.4939
**Newborn pregnancy intentions**					
Intended	864	31	7435	95	0.001
Unintended	884	24	4417	46	0.0035
**Marital status and Pregnancy intentions**					
Married and pregnancy was intended	696	24	6409	79	0.0008
Married and pregnancy was unintended	419	12	3266	27	0.0008
Single (Unmarried) and pregnancy was intended	167	6	952	14	0.1897
Single and pregnancy was unintended	463	12	1110	19	0.7940
**Parity**					
Primiparous	940	19	10,425	73	0.001
Multiparous (≥1)	1279	40	5380	116	0.039

CI-confidence interval. Primiparous – first time mothers, Multiparous – Given birth at least once previously

### Hazard ratios for neonatal mortalities among adolescent mothers compared to mothers 20–29 years old

Table 4 presents the findings from the cox proportional hazards regression model, showing overall hazard ratios (HR) for neonatal mortality among adolescent mothers, 15–19 years old, compared to mothers 20–29 years old. In model 1, when adjusted for sociodemographic factors and sex of the newborn, adjusted hazard ratio (aHR) for neonatal death among adolescents was almost twice as high, i.e. aHR 1.80 (95% CI 1.22–2.63). Neonatal mortalities among adolescent mothers occurred twice the rate per unit time compared to deaths among mothers 20–29 years old. Additional adjustments for antenatal care, place of birth, and postnatal care also generated a statistically significant HR, i.e. aHR 1.78 (95% CI 1.20–2.64). The results further show that being a female newborn, having more than four ANC visits, and at least one PNC visit during the first 28 days after birth were protective factors, while LBW was associated with higher hazard of death among adolescent-born neonates.

**Table 4.** Cox proportion hazards regression models showing hazard of death for neonates born to adolescents compared to those born to mothers, 20–29 years old in Kenya, Uganda, and Tanzania, 2014–2016.

Variable	Unadjusted HR	Model 1*(95%CI)	Model 2 (95%CI)	Model 3 (95%CI)
		aHR*	aHR**	aHR**
**Maternal age**				
Adolescent (≤ 19 years)	**1.98(1.36–2.87)**	**1.80(1.22–2.63)**	**1.78(1.20–2.64)**	**1.86(1.06–3.29)**
20-29 years old	1.00	1.00	1.00	1.00
**Place of residence**				
Rural	0.90(0.64–1.24)	0.98(0.69–1.41)	0.93(0.65–1.34)	0.82(0.49–1.38)
Urban	1.00	1.00	1.00	1.00
**Education level**				
No or primary education	1.32(1.00–1.72)	1.15(0.79–1.66)	1.10(0.76–1.60)	0.70(0.42–1.16)
Secondary or higher	1.00	1.00	1.00	1.00
**Wealth status**				
Poor	0.97(0.70–1.34)	0.75(0.53–1.07)	0.70(0.49–1.00)	0.75(0.43–1.30)
Middle and rich	1.00	1.00	1.00	1.00
**Marital status**				
Single/unmarried	1.57(1.11–2.22)	1.41 (0.98–2.01)	1.41(0.98–2.02)	––
Married	1.00	1.00	1.00	––
Newborn pregnancy intended*				
Unintended	0.85(0.65–1.11)	0.75(0.52–1.10)	0.72(0.49–1.05)	––
Intended	1.00	1.00	1.00	––
**Antenatal care (ANC) visits**				
<4	**1.45(1.06–1.97)**		**1.40(1.02–1.93)**	**1.73(1.08–2.77)**
≥4	1.00		1.00	1.00
**Place of delivery**				
Home	0.94(0.67–1.32)		1.01(0.70–1.46)	0.74(0.29–1.85)
Health facility	1.00		1.00	1.00
**Postnatal care (PNC) visit(s) within 28 days after birth**				
No	**1.76(1.16–2.66)**		**1.69(1.11–2.56)**	**2.78(1.49–5.20)**
Yes	1.00		1.00	1.00
**Sex of child**				
Female	**0.69(0.49–0.92)**	**0.67(0.49–0.93)**	**0.66(0.48–0.91)**	**0.58(0.34–0.95)**
Male	1.00	1.00	1.00	1.00
**Low birthweight**				
Yes	**3.57(2.49–5.14)**			**4.43(2.76–7.11)**
No	1.00			

Model 1. Adjusted for sociodemographic factors, pregnancy intentions and sex of child

Model 2. Adjusted for all model 1 covariates and ANC, PNC, and Place of delivery

Model 3. Adjusted for all covariates in model 1 and model 2 (except marital status and pregnancy intentions) and low birthweight

*Marital status was used to determine HR in all models 1 and 2 in the absence of ‘Newborn pregnancy intended’ variable and newborn pregnancy intended was added to the model in the absence of variable ‘Marital status’ due to collinearity

**Bolded** results are statistically significant (95% confidence interval (CI)). LBW – low birthweight, NBW

### Kaplan-Meier survival curves depicting survival over 28 days by maternal age and log rank estimates

**Figure 1.**
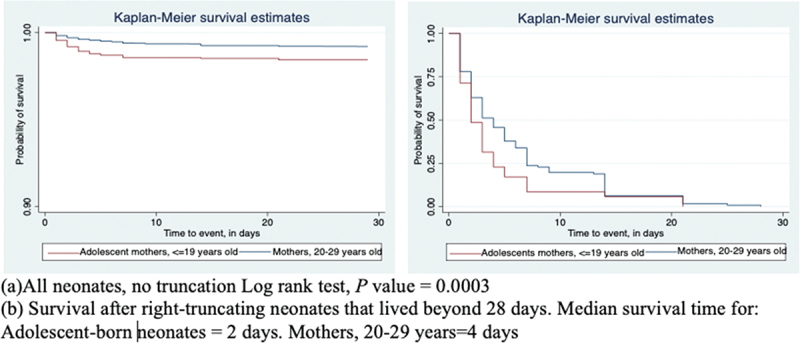
**a-b**. Kaplan-Meier survival functions for neonates born to adolescent mothers (≤ 19 years old) and those born to mothers aged 20–29 years in Kenya, Uganda and Tanzania, 2014–2016.

Figure 1(a-b) is Kaplan Meier survival curves showing a statistically significant (log rank, chi square, one degree of freedom, *X*^2^(1) = 13.27, and *p*=0.0003) difference in neonatal survival by maternal age for neonates born to adolescent mothers compared to those born to mothers 20–29 years old.

**Figure 2.**
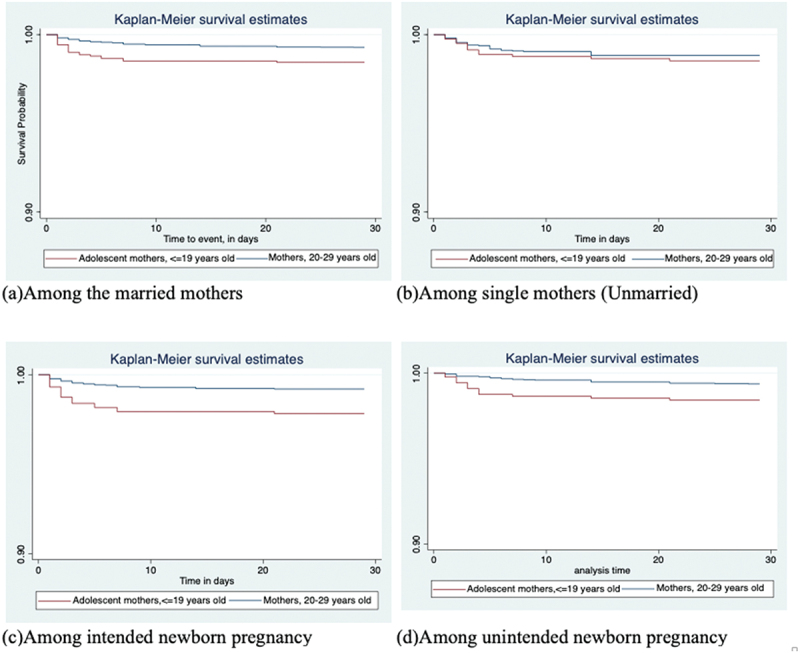
**a-d**. Kaplan-Meier survival curves by maternal age-group, stratified by marital status, (a)-(b) or Pregnancy intentions (c)-(d).

**Figure 3.**
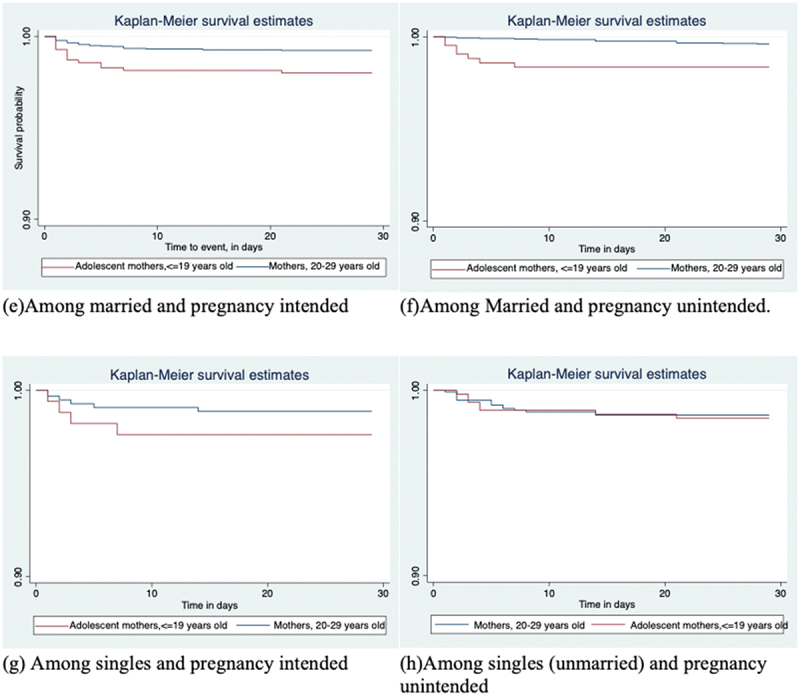
**e-f**. Kaplan-Meier survival curves by maternal age-group, stratified by marital status and Pregnancy intentions for adolescent-born neonates versus neonates born to mothers 20–29 years old in Kenya, Uganda, and Tanzania, 2014–2016.

Similar, Figure 2(a-d), Figure 3(e-h) and Table 3 show that survival time associated with neonatal deaths was significantly shorter for mortalities among adolescent mothers than that of their corresponding older mothers for all stratified analyses except among single (unmarried) mothers (*p*=0.4939), irrespective of their pregnancy intentions. However, the number of mothers in the single category was relatively small.

### Test for proportional-hazards assumption

**Figure 4.**
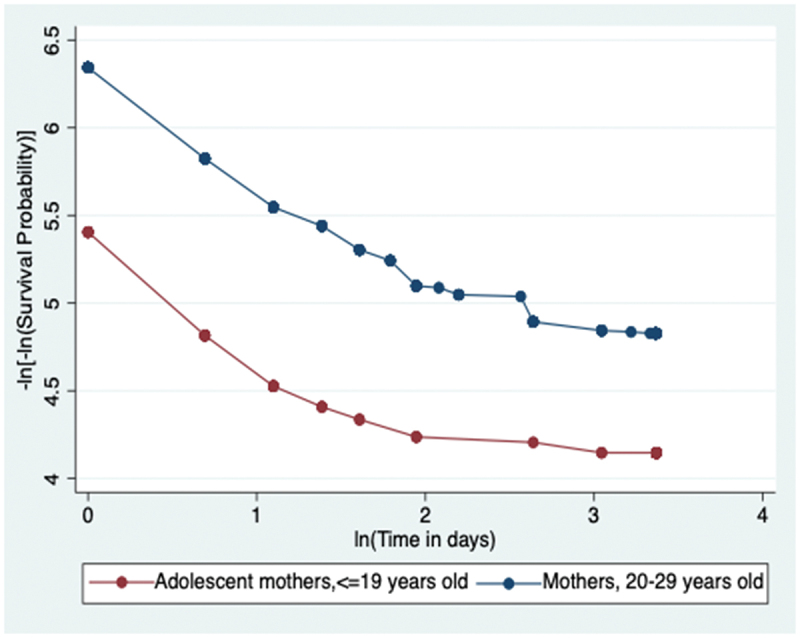
Graphical assessment of proportional-hazards assumption for the Cox proportion hazards regression models comparing hazard of neonatal death among neonates born to adolescents compared to those born to mothers, 20–29 years old in Kenya, Uganda, and Tanzania, 2014–2016.

The p-value of the global Schoenfeld test is 0.4128, not statistically significant and the graphical representation in Figure 4 (in the appendix) is the log-log transformation of the overall survival function; the two curves for the two age-groups of mothers are roughly parallel without meeting or intersecting. Both the non-statistically significant p-value of the global test and the roughly parallel curves of the log-log transformation indicate that the proportional hazards assumption is satisfied.

### Effect of marital status or pregnancy intentions on the hazard ratios for neonatal deaths by maternal age

Table 5 shows adjusted hazard ratios for neonates born to adolescents versus neonates born to mothers 20–29 years old, stratified by marital status or pregnancy intentions. The aHR for neonatal deaths among adolescents was more than twofold higher compared to those born to older mothers, among all married mothers versus unmarried, aHR 2.20 (95% CI 1.37–3.52) and among all adolescent mothers whose pregnancy was intended, aHR 2.84 (1.67–4.81) or unintended, aHR 2.51 (1.32–4.79). The aHR among the unmarried was not statistically significant, aHR 1.13 (95% CI 0.59–2.27)

**Table 5.** Adjusted hazard ratios (aHR)* for neonatal mortality among adolescent mothers compared to mothers, 20–29 years old in Kenya, Uganda, and Tanzania, 2014–2016, stratified by marital status or ^†^pregnancy intentions.

Variable	Model 1, aHR	Model 2, aHR	Model 3, aHR	Model 4, aHR
Adolescent mothers, ≤19 years old	2.20 (1.37–3.52)	1.13(0.59–2.27)	2.84(1.67–4.81)	2.51(1.32–4.79)
Mothers, 20–29 years old	1.00	1.00	1.00	1.00
Model 1- Among married mothersModel 2- Among unmarried mothers			Model 3-Neonates from intended pregnancyModel 4-Neonates from unintended pregnancy	

*Adjusted for sociodemographic factors and maternal care variables (antenatal and postnatal attendance and place of delivery and PNC)

^†^Whether or not the neonate pregnancy was intended. LBW was not adjusted for due to insufficient data in various strata.

In Table 6, further stratification by combined marital status and newborn pregnancy intentions indicate a fourfold higher hazard of neonatal death for married adolescent mothers whose pregnancy was unintended, aHR 4.08 (95% CI 1.62–10.31), compared to corresponding older mothers aged 20–29 years. HR among married adolescent mothers whose pregnancy was intended was about three times higher compared to their older counterparts, aHR 2.86 (95% CI 1.55–5.26). However, HR was higher but not statistically significant among unmarried adolescent mothers with or without pregnancy intentions. HR among primiparous adolescent mothers, compared to their older counterparts aged 20–29 years, was much higher compared to HR among multiparous adolescent mothers when compared to their older multiparous counterparts.

**Table 6.** Adjusted hazard ratios (aHR)* for neonatal deaths among adolescent mothers compared to mothers, 20–29 years old in Kenya, Uganda, and Tanzania, 2014–2016, stratified by marital status and ^†^pregnancy intentions, both overall and among primi- and multi-parous mothers.

Overall				
Variable	Model 1, AHR	Model 2, AHR	Model 3, AHR**	Model 4, AHR**
**Maternal age**				
Adolescent mothers, 15–19 years old	2.86(1.55–5.26)	4.08(1.62–10.31)	1.89(0.59–6.08)	1.13(0.46–2.80)
Mothers, 20–29 years old	1.00	1.00	1.00	1.00
**Among primiparous only (First time mothers)**				
Adolescent mothers, ≤19 years old	4.32(1.41–13.27)	6.48(1.37–30.71)	–	1.56(0.39–6.09)
Mothers, 20–29 years old	1.00	1.00	–	1.00
**Among multiparous only (Given birth at least once previously)**				
Adolescent mothers, ≤19 years old	1.84(0.89–3.80)	2.43(0.75–7.98)	–	0.63(0.19–2.11)
Mothers, 20–29 years old	1.00	1.00	–	1.00
Model 1- Among married mothers and newborn from intended pregnancy Model 2- Among married mothers and newborn from unintended pregnancy			Model 3- Among unmarried mothers and newborn from intended pregnancy Model 4- Among unmarried mothers and newborn was unintended pregnancy	

*Adjusted for sociodemographic factors and maternal care variables (antenatal and postnatal attendance and place of delivery)

^†^Whether or not the neonate pregnancy was intended. Birth weight was not adjusted for due to insufficient data in the various strata.

** Insufficient mortality data among unmarried (single) mothers with intended pregnancies hindered plausible analysis

## Discussion

In this study we examined the survival patterns among neonates born to adolescent mothers, 15–19 years, as compared to older mothers aged 20–29 years and the effect of pregnancy intentions and marital status on time-to-death patterns in Kenya, Uganda, and Tanzania. Overall, after adjusting for confounders, the hazard of death among neonates born to adolescent mothers was 1.8 times higher (almost twice the rate per unit time) compared to those born to mothers 20–29 years old. Considering only mothers who had unintended pregnancies, the hazard of neonatal deaths among adolescent mothers was over 2.5-fold higher than that among older mothers. The highest (four-fold) hazard of neonatal death was among adolescent mothers who had unintended pregnancies in marital union. Insufficient data hindered further comparative analysis among unmarried mothers. Joint estimates for the three countries show that over 50% of adolescent pregnancies were unintended, although Tanzania had slightly lower (39%) unintended pregnancies among adolescents.

This study is probably the first of its kind in East Africa. Our overall finding of HR 1.8 for hazard of mortality among neonates born to adolescent mothers is comparable to findings of a nationwide study conducted in Nigeria, a similar setting, by Akinyemi et al. that found hazard ratios of 1.75 and 1.5 for its 2008 analytical models, comparing neonatal deaths among adolescent mothers to mothers 20–35 years old [52]. Other studies across the globe have reported comparable higher risks of neonatal death among neonates born to adolescent mothers [5,19,20,53,54]. Furthermore, the USA 2020 national report indicated highest NMR among adolescent mothers compared to older mothers [55]. This is corroborated by our finding, demonstrating that NMR was twice as high among teenage mothers compared to mothers 20–29 years old (26.6 versus 12.0 deaths per 1000 live births). Adolescent pregnancy and associated higher neonatal mortality, as well as maternal deaths, are more prevalent in LMIC, although they are global health burdens affecting even certain high-income countries [56,57].

### Possible interventions for pregnant adolescent mothers

It is widely agreed that the focus of interventions to eliminate higher neonatal deaths among adolescent mothers should be geared chiefly towards preventing adolescent pregnancies through use of contraceptives and education. However, every pregnant adolescent in Kenya, Uganda and Tanzania ought to be handled with a sense of high-risk status that necessitates emergency preparedness at all stages of care and in all maternity centers. Identification of such a pregnancy at community level could receive support from community health workers (CHW) to encourage parental support, early initiation of antenatal visit and follow-up to health facility delivery, and postnatal care attendance by both health facility personnel and CHW.

Similarly, policy regulations necessitating all adolescent antenatal and postnatal care and childbirth to be conducted by skilled personnel in a well-equipped health facility ought to be introduced. Furthermore, parents or guardians of pregnant adolescents could be sensitized by CHW through brief educational programs to sensitize them on the higher risk factors associated with such pregnancies and on how they could best support the child mothers. These programs could be instituted and funded by the governments of respective countries.

### Other intermediate risk factors

In contrast, a study in rural Nepal found no significant difference in neonatal mortality among adolescent mothers, compared to mothers aged 20–24 years, after adjusting for a range of variables including birthweight and preterm birth [58]. The study, however, found much higher NMR associated with LBW and preterm births among adolescent mothers than mothers 20–24 years old [58]. Although our study could not examine possible physiological pathways leading to higher neonatal deaths among adolescents, we found higher hazard of neonatal deaths among adolescents even after adjusting for birthweight. Nonetheless, a 2021 Lancet study of a population-based cohort in England found that younger mothers (age<20 years) and older mothers (age>37 years) had lowest birthweight newborns [59]. The study also found that LBW newborns were prevalent in deprived areas, indicating that undernutrition, as well as adolescent age and much older maternal age are potential pathways to LBW. Moreover, two current systematic reviews reported that LBW is common among adolescents and is associated with neonatal deaths [60,61]. LBW and preterm are known to be leading causes of neonatal deaths in South Asia and sub-Saharan Africa [44]. Thus, this largely explains the lower survival rate of neonates born to adolescents in our study. Preconception interventions aimed to reduce risky sexual behaviors during pre-pregnancy, as suggested by Hemsing et al. [62], could play a critical role given the interrelation between low birthweight and adolescent pregnancy. Already existing policies in East Africa that promote institutional delivery and the upgrading of health facilities could also be implemented and enforced to create a greater preparedness for all adolescent births. Additionally, adequate care preparations for LBW newborns, such as artificial respirators and nutritional necessities, could be availed for all adolescent childbirths [63]. Furthermore, programs to improve parenting efficacy during the neonatal period should be considered for adolescent mothers in the three East African countries [64].

### *Complexity of teen pregnancy burden in East* Africa

Our findings highlight the complexity of the challenge to reduce preventable neonatal deaths in East Africa. To achieve Agenda 2030, target 3.2 that aims to drastically reduce neonatal death rates [30], focus on adolescents will have to be highly prioritized in East Africa. With 21% teen pregnancy prevalence and close to 27 deaths per 1000 live births in Kenya, Uganda and Tanzania [13], neonates born to adolescents contribute a substantial proportion of total neonatal deaths. Preventing these deaths requires much more than just access to obstetric healthcare services. Especially as adolescent age is a critical developmental stage, biologically, socially, and mentally [57,59,65].

Over half of adolescents in our study had unintended pregnancy and in our stratified findings among married mothers, the hazard of death doubled to fourfold for neonates born to adolescents from unintended pregnancies. Akinyemi et al. also found being married had significantly lower (about 50%) hazard ratios for neonatal deaths, although their findings included all mothers of reproductive age, e.g. 15–49 years [53]. Further, 80% of our sample was married, and while studies reveal that the number one cause of adolescent pregnancy is marriage [1,10,11,13], it is also very plausible to hypothesize from our findings that unintended pregnancy is a major risk factor for adolescent marriage. A study reported that unlike South Asia where adolescent marriages are planned in advance, in SSA [66], unintended pregnancy precede ‘unplanned’ adolescent marriages [66,67].

Consequently, in SSA, adolescent marriages are prone to school dropout, poverty [68], HIV infections [69], intimate partner violence [70,71], and associated negative mental health impacts [72] that in turn lead to poor neonatal outcomes [73]. Also, studies have reported mistreatment and discrimination of adolescent mothers during births which could be a deterring factor to seek care even during pregnancy [74].

### Existing policy guidelines

Efforts to reduce adolescent pregnancies in East Africa have not yielded any marked outcomes in recent years. The Uganda adolescent health policy of 2004 had its target to halve the proportion of women bearing a child before 20 years of age to 31% [75]. Yet, by 2015, over 51% of women still had their first-born before 20 years of age [76]. Similar statistics are reported in Kenya [77] and Tanzania [78]. The challenge seems to be the implementation of the guidelines. In Uganda, the revised 2020 guidelines for prevention of teenage pregnancy in schools provide a comprehensive outline of the roles of key actors that include schools, involving teaching and enforcing pregnancy prevention measures, such as no sexual relationships at school [79]. Additionally, retaining and supporting pregnant adolescents in school should be strongly supported [79]. In Kenya, the national adolescent sexual and reproductive health policy has detailed a multisectoral approach [80]. In Tanzania, the national adolescent strategy outlines a comprehensive action plan for 2018–2022 [81]. The need to effectively implement the sexual and reproductive education proposed by all three guidelines cannot be overemphasized and sociocultural norms highlighted as a major hindrance should not be underestimated.

### Health education and modern contraceptive use

Our findings provide a vital rationale to synergize advocacy efforts at both national level and in the East African community to enable reduction of adolescent pregnancies and related neonatal deaths. Creative efforts to educate both girls and boys on the sociocultural norms that impact their lives, promoting those which are improving equality and access to education, are required to maximize protective effects of girl education and encourage and foster behavior change. Although many policies exist in East Africa, sexual and reproductive health and rights education (SRHR) is not practically emphasized in most schools – except for sexual and reproductive physiology and HIV prevention [82–85]. With the high levels of unintended pregnancies and adolescent marriages, we suggest that well instituted, regular, and expert guided SRHR talks with parents and adolescents be continuously conducted in communities and schools to raise awareness and improve access to contraceptives. Training and engaging local youthful role models as health educators would ensure sustainability. Further, there is promising evidence regarding the positive relationship between including young people in health projects and identifying need-based and acceptable solutions [86,87]. Health research and interventions may therefore consider the ways in which youths are involved to ensure aspects of inclusion, representation and participation. Further research could examine the proportion of undocumented, unintended pregnancies that end up in safe or unsafe abortion that could have been avoided if effective contraceptives were freely available and used by adolescents [88,89].

### Methodological considerations

Our large dataset combining three nationally representative data enables plausible analysis and valid findings for the three highly populated East African countries where very few population-based studies have been conducted on survival pattern among adolescent-born neonates. The retrospective nature of the cross-sectional data collection by the DHS could have been affected by recall bias. Nevertheless, childbirth is a special occurrence that is not forgettable even over a long period, and by using most recent live birth within the last five years, our results substantially reflect the true maternal and neonatal situation in East Africa. Many mothers also have records of birth certificates for their last-born children. Further, response biases, such as social desirability bias, were also minimized by thorough training of the DHS interviewers and the wealth of knowledge and practice DHS has used to improve surveys over the years [90].

A good proportion of low birthweight (lbw) newborns are also preterm [8], however, we could not ascertain whether the neonates in this study were preterm or full-term babies. Perhaps in addition to lbw, including gestation age at birth in the study may have provided more light on the findings.

## Conclusion

In summary, neonatal deaths among adolescent mothers, 15–19 years old, occurred about two times faster, compared to deaths among neonates born to older mothers 20–29 years old in the three East African countries of Uganda, Kenya and Tanzania. This hazard ratio for neonatal deaths doubled to four times among married adolescent mothers with unintended pregnancies, and in addition tripled to six times among first-time mothers (primiparous).

The findings in this study are critical in that they reveal how unintended pregnancies and deaths of neonates born to adolescent mothers contribute substantially to preventable neonatal deaths in East Africa. We propose increased support for sexual and reproductive health and rights (SRHR) education in schools and communities and improved access and use of contraceptives among sexually active adolescents. Young people play a key role in identifying their own needs, priorities and possible solutions, which calls for strategic partnerships with youths themselves in both health research and interventions. Novel efforts that address sociocultural norms to reduce adolescent pregnancies or marriage should be strongly supported. Similarly, stringent regulations requiring all adolescent obstetric care to be conducted by skilled personnel, sensitized on attending to young people’s needs, could be considered in Kenya, Uganda, and Tanzania – not the least in rural areas where access is even more limited. Noting the weak implementation of policies, Kenya, Uganda and Tanzania should strongly consider comprehensive implementation of existing adolescent health policies as well as monitor and evaluate them to prevent unintended pregnancies. Further, retention and reentry of adolescent mothers-to-be or mothers in school is vital. Regulations requiring adolescents’ obstetric care to be conducted by skilled personnel could be introduced and implemented.

## Paper context

Unintended pregnancies and associated neonatal deaths disproportionately impact babies born to adolescent girls in low – and lower-middle – income countries. Our study examined and revealed the survival patterns of neonates born to adolescents in Kenya, Uganda and Tanzania and the effects of related factors such as marital status and pregnancy intentions. To reduce neonatal mortality burdens in low-income settings, further research and key interventions aimed at prevention of adolescent marriages and unintended pregnancies are critical for the realization of reduced newborn deaths.
